# Internet-based cognitive behavioural therapy (iCBT) for perinatal anxiety and depression versus treatment as usual: study protocol for two randomised controlled trials

**DOI:** 10.1186/s13063-017-2422-5

**Published:** 2018-01-22

**Authors:** Siobhan A. Loughnan, Jill M. Newby, Hila Haskelberg, Alison Mahoney, Natalie Kladnitski, Jessica Smith, Emma Black, Christopher Holt, Jeannette Milgrom, Marie-Paule Austin, Gavin Andrews

**Affiliations:** 1 Clinical Research Unit for Anxiety and Depression (CRUfAD), UNSW School of Psychiatry at St Vincent’s Hospital, Level 4, O’Brien Centre, St Vincent’s Hospital, 394 Victoria Street, Sydney, NSW 2010 Australia; 2 School of Psychology, Faculty of Science, UNSW Sydney, 1302 Mathews Building, Kensington, NSW 2052 Australia; 3 Perinatal & Women’s Mental Health Unit, c/o St John of God Hospital, 13 Grantham Street, Burwood, NSW 2134 Australia; 4 Parent-Infant Research Institute (PIRI) and Melbourne School of Psychological Science, Heidelberg Repatriation Hospital, 300 Waterdale Road, Heidelberg West, VIC 3081 Australia

**Keywords:** Perinatal, Antenatal, Postpartum, Pregnancy, Internet, Online, Cognitive behavioural therapy, Treatment, Depression, Anxiety

## Abstract

**Background:**

We aimed to evaluate the acceptability and efficacy of two brief, Internet-delivered cognitive behavioural therapy interventions—*MUMentum Pregnancy* (study 1) and *MUMentum Postnatal* (study 2)—in reducing maternal symptoms of anxiety, depression and overall psychological distress compared to usual care in the perinatal period.

**Methods/Design:**

Women who are pregnant (study 1) or < 12 months postpartum (study 2) with current clinically elevated symptoms of anxiety and/or depression according to validated self-report measures, will be recruited via the research arm of a not-for-profit clinical and research unit in Australia and randomised to the intervention group or treatment as usual control group. The minimum sample size for each study (alpha 0.05; power 0.80 for a *g* of 0.80) was identified as 50 with at least 10% more to be recruited to account for expected attrition. The co-primary outcome measures are the Patient Health Questionnaire 9-item scale and Generalised Anxiety Disorder 7-item scale to measure depression and anxiety symptom severity, respectively, and will be administered at the following primary time-points: baseline; post treatment; and at one-month follow-up. Psychological distress will be measured according to the Kessler-10 psychological distress scale at each primary time-point and will also be completed before each lesson for those in the intervention group. The total trial period nine weeks for study 1 and 11 weeks for study 2. Program efficacy will be determined using intent-to-treat mixed models. Maintenance of gains will be assessed at one-month follow-up.

**Discussion:**

The current randomised controlled trial seeks to extend the literature by evaluating the efficacy of a self-help intervention for women in the perinatal period. If efficacious, the *MUMentum* programs have the potential to be easily disseminated via https://thiswayup.org.au/ to large numbers of women across Australia as an intervention for women screening positive for anxiety, depressive or distress symptoms during pregnancy or postpartum.

**Trial registration:**

Australian New Zealand Clinical Trials Registry, ACTRN12616000560493; ACTRN12616000559415. Registered on 2nd May 2016.

**Electronic supplementary material:**

The online version of this article 10.1186/s13063-017-2422-5) contains supplementary material, which is available to authorized users.

## Background

Maternal anxiety and depression is common during the perinatal period (i.e. pregnancy through to 12 months postpartum) [1, 2]. Prevalence rates estimate up to 20% of women will experience clinically significant symptoms of anxiety or depression during this time, with symptoms often co-occurring [3, 4]. Yet fewer than half of these women will seek help or receive evidence-based treatment [1, 5, 6]. Women face many structural and attitudinal barriers to accessing and engaging with treatment including lack of time, cost, geographical distance to services, transport issues, and practical difficulties with childcare [5, 7–9]. Women are also often unwilling to disclose problems associated with motherhood to partners, family and health professionals due to perceived social stigma (e.g. pressure to minimise emotional difficulties as a mother) [1, 7].

In the absence of routine screening in community-based and hospital-based settings, clinically significant symptoms of perinatal anxiety and depression remain under-detected and undertreated [2, 10]. This is a significant problem given anxiety and depression are associated with short-term and long-term adverse outcomes for the mother and infant (e.g. increased risk of obstetrical complications, poor birth outcomes and child developmental problems; detrimental to the mother–infant relationship) [11–14]. If left untreated, perinatal anxiety and depression can also lead to a chronic or recurring course of symptoms throughout the mother’s life [6]. Developing evidence-based treatments that are acceptable and easily accessible for perinatal women (e.g. no referral required; minimal logistical issues) is an important area of focus that warrants attention.

One approach to help increase access to treatment is through the use of Internet-delivered psychological interventions, particularly cognitive behavioural therapy (CBT). In comparison to face-to face treatment, Internet-delivered CBT (iCBT) is scalable (e.g. does not rely on clinician time and resources in order to be disseminated at a population level), available at a reduced cost, more accessible especially for rural and remote patients, and can be completed at home offering increased convenience to the patient [15, 16].

Preliminary research suggests that iCBT is efficacious for women with depression in the perinatal period. One randomised controlled trial (RCT) [17] evaluated a therapist-assisted six-session iCBT intervention, *MumMoodBooster*, for women with a clinical diagnosis of postpartum depression. This multimedia intervention included weekly therapist coaching via telephone. Post treatment, depression symptom severity scores were significantly lower for the intervention group compared to the treatment as usual (TAU) control condition (Cohen’s *d* = 0.83), with 79% of women in the intervention group no longer meeting diagnostic criteria for depression at 12 weeks, compared to 18% in the TAU control condition. Adherence and patient satisfaction with the program was also high. Pugh et al. [18] conducted an RCT to evaluate a seven-session iCBT intervention, *Maternal Depression Online*, for women with symptoms of subthreshold and clinical postpartum depression. This multimedia intervention included weekly therapist support and encouragement via email. For those in the iCBT group, symptoms of postpartum depression were significantly reduced from pre to post treatment and maintained at four-week follow-up compared to those participants in the waitlist control condition. Acceptability of the program and therapeutic alliance was reported as high.

Only one RCT [19] has evaluated an antenatal iCBT intervention for women with a clinical diagnosis of depression during pregnancy. The ten-week guided intervention, delivered as an adjunct intervention in addition to usual antenatal care, was supported by a CBT-trained therapist who provided regular feedback and encouragement. Compared to the TAU control group, depression scores significantly reduced from pre to post treatment for the iCBT group, with a large between-groups effect (Hedge’s *g* = 1.21). Treatment credibility and satisfaction with the intervention was high.

As this area of research is still in its early stages, there are significant gaps in the literature that need to be addressed. Although perinatal anxiety is reported to have similar prevalence rates to perinatal depression [3], and the two disorders are often highly co-morbid [20], no Internet-delivered interventions have targeted the reduction of maternal anxiety symptoms alone or anxiety symptoms co-morbid with depression. Given that anxiety and depression are thought to share similar aetiological and maintenance processes [21–23], transdiagnostic iCBT, which targets these shared factors, is a promising avenue for the treatment of anxiety or depression in perinatal women. Transdiagnostic iCBT interventions have the potential to teach treatment principles that can be generalised across anxiety and depressive diagnoses [24], as well as educate women about symptoms of both anxiety and depression regardless of whether they meet clinical levels. It has also demonstrated efficacy in treating primary and co-morbid anxiety and depression in the general adult population [25, 26].

ICBT for women in the antenatal period has also received limited attention, with only one study having evaluated an iCBT intervention for antenatal depression [19]. This is surprising given that antenatal anxiety and depression are two of the strongest predictors of postpartum depression [27, 28]. In addition, research to date has focused only on the evaluation of six- to seven-session ‘guided’ interventions for perinatal depression which are supported by trained therapists. No studies have evaluated brief (i.e. < 6 sessions) or ‘unguided’ (i.e. no therapist assistance) interventions for anxiety and/or depression in either the antenatal or postpartum period.

## Objectives

The primary aim of the proposed studies is to test the acceptability and the efficacy of the iCBT *MUMentum* programs—*MUMentum Pregnancy* (study 1) and *MUMentum Postnatal* (study 2)—in reducing symptoms of anxiety, depression and overall psychological distress compared to usual care in the perinatal period. We hypothesise that the two iCBT interventions will significantly reduce symptoms of distress, anxiety and depression and will be significantly more effective than usual care. The secondary aim is to explore the effects of the iCBT programs on mother–infant bonding, parenting confidence and maternal quality of life. We hypothesise that the iCBT programs will lead to significant improvements in these three factors compared with usual care.

## Methods

### Design

Both studies are parallel group, CONSORT-revised 2010 compliant [29] superiority RCTs of iCBT program vs TAU control. Study 1 is a nine-week trial, including baseline data collection, four-week intervention period, one-week post-treatment evaluation and one-month follow-up. Study 2 is an 11-week trial, including baseline data collection, six-week intervention period, one-week post-treatment evaluation and one-month follow-up. The current trial protocol has been approved by the Human Research Ethics Committee of St. Vincent’s Hospital, Sydney (HREC/16/SVH/63). Both studies are registered with the Australian New Zealand Clinical Trials Registry (study 1: ACTRN12616000560493; study 2: ACTRN12616000559415).

### Study setting

The Clinical Research Unit for Anxiety and Depression (CRUfAD) is a non-profit joint initiative of St. Vincent’s Hospital, Sydney and the University of New South Wales (UNSW), School of Psychiatry, Sydney, Australia. CRUfAD specialises in the development, evaluation and dissemination of evidence-base CBT programs via https://thiswayup.org.au/. These RCTs will be conducted within CRUfAD’s clinical research arm, the Virtual Clinic (https://virtualclinic.org.au/). The mode of Internet recruitment and delivery enables potential participants from all Australian states to apply for enrolment in the current trials, with no face-to-face contact required.

### Participants and recruitment

Participants will be recruited through flyers (located in public maternity hospitals in Sydney and GP clinics Australia-wide), paid Internet advertising (e.g. Facebook via THIS WAY UP Clinic) and word of mouth (e.g. midwives, general practitioners [GPs], obstetricians; conference presentations).

#### Inclusion criteria

Both studies will recruit adult women aged > 18 years, who are 13–30 weeks pregnant (study 1) or within 12 months postpartum (study 2). Applicants will meet clinical cut-off scores (>9) for anxiety and/or depressive symptoms as measured on the Generalised Anxiety Disorder 7-item version (GAD-7) [30] and Patient Health Questionnaire 9-item version (PHQ-9) [31]. Additional inclusion criteria are as follows: computer and Internet access; Australian resident; fluent in written and spoken English; willing to provide name, phone number and address, and to provide the name and address of their GP.

#### Exclusion criteria

Both studies will exclude applicants who report current substance abuse or dependence; a diagnosis of schizophrenia or bipolar disorder; current use of benzodiazepines or who have recently started psychological therapy for depression/anxiety (<4 weeks) or medication for depression/anxiety (<8 weeks). Applicants reporting current suicidality (indicated by a score of 3 to item 9 of the PHQ-9; and/or a score of 2 or 3 to item 9 of the Beck Depression Inventory, version 2 (BDI-II) [32]; and/or severe depression (indicated by score ≥ 23 on PHQ-9) [31] will also be excluded. Applicants that self-report occasional thoughts of suicidality (indicated by a score of 1 or 2 to item 9 of the PHQ-9; and/or score of 1 to item 9 of the BDI-II) will be contacted by the study clinician for a risk assessment telephone interview and if not suitable (i.e. endorse current suicidality) will be excluded.

### Procedures

#### Online application and psychological assessment

**Fig. 1:**
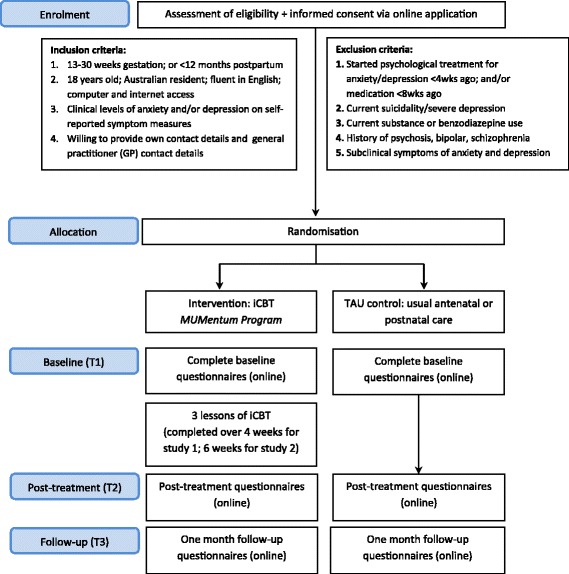
*Flowchart* illustrating enrolment and study procedure

The enrolment and study procedure are outlined in Fig. 1. All women apply online through the Virtual Clinic website (https://virtualclinic.org.au/) and are required to provide electronic informed consent, personal contact details and GP contact details. Applicants will then complete online screening questionnaires to determine suitability. There are three outcomes for all online applications: (1) applicants that do not meet inclusion criteria will be notified on-screen and via email that they are not suitable; (2) applicants who meet inclusion criteria yet indicate possible risk of suicidality, will be ‘pending’ until a telephone risk interview (by the supervising clinician) can be conducted to determine suitability for inclusion into the study; and (3) applicants that meet all inclusion criteria and indicate no risk of suicidality will proceed to randomisation.

All applicants that do not meet inclusion criteria will receive information on alternative services and encouraged to discuss their symptoms with their GP. All GPs of applicants that are excluded on severe depression and/or current suicidality criteria will be notified via letter that their patient applied for this study and was excluded on this basis.

#### Randomisation and allocation procedures

Online applications meeting the study’s inclusion criteria will be randomised to either iCBT or a TAU control group according to a 1:1 ratio within blocks of 20. Because the two trials were conducted using different software, the randomisation procedure differs slightly between the studies.

For study 1, the allocation sequence will be generated by an independent person not involved in the study, using a random number generator (https://www.random.org/). The numbers corresponding to the treatment group (1 = iCBT) or the control group (2 = TAU) will be placed in sequentially numbered, sealed opaque envelopes. The randomisation envelopes will then be assigned manually (by the study coordinator) to the successful online application in order of application date. Once matched, the group allocation will be revealed and participants will be notified of their group allocation by e-mail.

For study 2, which will be conducted in an updated Virtual Clinic web system,^1^ the randomisation sequence will be electronically uploaded to the server by an independent person not involved in the study. Successful applicants will be notified via e-mail of their acceptance into the study and automatically randomised to treatment group (1 = CBT) or control group (2 = TAU).

**Fig. 2:**
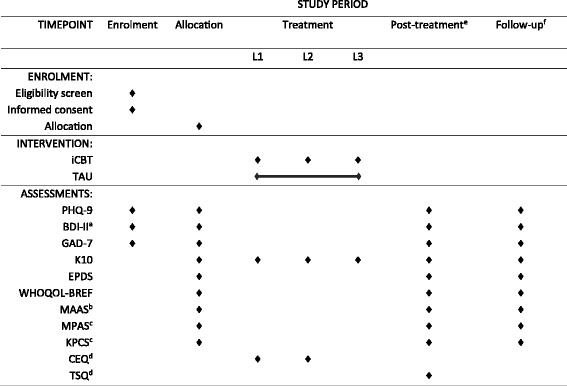
Schedule of enrolment, interventions and assessments

Once randomised, participants and research personnel will not be blinded to group condition. Participants in the intervention group and control group will login to https://virtualclinic.org.au/ and complete baseline (pre-treatment) questionnaires. The three primary data collection points for study participants in iCBT group and TAU group are as follows: baseline (pre-treatment), post treatment, and at one-month follow-up (see Fig. 2). Participants will receive notifications and reminders to complete questionnaires via computer-generated emails, SMS and follow-up phone calls from the research team. We will track reasons for non-adherence (e.g. lost to follow-up). As this is an unguided program, participant enquiries (e.g. technical issues) will be responded to by research technicians; no coaching or support with program content will be provided.

Participants allocated to the iCBT group will receive access to the *MUMentum* program via their Virtual Clinic account. All three lessons are required to be completed within the active treatment period, with one lesson released every seven days. The treatment period for study 1 is four weeks, which allows participants up to one week to complete each lesson and an additional week for revision. For study 2, the treatment period is six weeks, to allow participants up to two weeks to complete each lesson (without a revision week). Time to complete lessons was increased for study 2 as it was predicted this would be more suitable for postnatal women who are time-poor and adjusting to and caring for a newborn. There will be a five-day lockout period between lessons to ensure participants spend time revising and implementing the lesson material before moving onto the next lesson. At the beginning of each lesson, participants will complete the Kessler-10 psychological distress scale (K10) [33] as a brief measure of psychological distress, as well as two brief questions relating to time spent completing the previous lesson and homework activities. Participants will be notified of new lessons and reminded to stay on schedule via email and SMS reminders. One week after the active treatment period ends, participants will receive post-treatment questionnaires, with follow-up questionnaires completed four weeks later.

Participants allocated to the TAU group will receive access to questionnaires at each primary data collection point (i.e. baseline, post treatment, follow-up) via their Virtual Clinic account. The total trial period is nine weeks for study 1 and 11 weeks for study 2. As this is a ‘treatment as usual’ controlled study, all participants in the control group will have access to their usual care (i.e. continuation of their current maternity care). Information will be collected from participants regarding usual care. For ethical reasons, participants in the TAU group will be offered access to both the *MUMentum Pregnancy* and *MUMentum Postnatal* iCBT programs at conclusion of the study period.

#### Safety protocol

The *MUMentum* programs are unguided (i.e. no clinician support regarding program content) interventions with risk monitoring. Computer-generated alert emails will be sent to participants at completion of questionnaires if there is an increase in psychological distress as measured on the K10 before each lesson (i.e. one standard deviation increase from previous score and/or total score ≥ 30), depression as measured on the PHQ-9 at primary data collection points (i.e. one standard deviation increase from previous score and/or total score ≥ 23) or suicidality scores as measured on item 9 of PHQ-9, item 10 of EPDS, and item 9 on BDI-II at primary data collection points (i.e. score > 1). Alert emails will notify participants of increases in scores, where to seek additional support (e.g. GP) and emergency contact numbers. For both studies, alert emails will be monitored by the study clinician (i.e. psychiatry registrar; clinical psychologist) and contact will be made with the participant only if additional action to the automated alert email is required (e.g. phone call). Each participant’s GP will be notified via letter that their patient is enrolled in our research study and will be contacted further if safety concerns for their patient arise during the trial or their patient is withdrawn.

#### Primary outcomes measures

All measures will be collected at specified time-points throughout the trial as outlined in Fig. 2. As this study is evaluating a transdiagnostic iCBT program, the co-primary outcomes will be anxiety and depression symptom severity.

*Patient Health Questionnaire 9-item scale* (PHQ-9) [31]. The PHQ-9 is a nine-item self-report measure of depressive symptoms experienced over the past two weeks. The PHQ-9 is based on the Diagnostic Statistical Manual, fourth edition (DSM-IV) [34] diagnostic criteria for major depressive disorder (MDD). Participants rate the frequency of symptoms (e.g. ‘Feeling down, depressed, or hopeless’) on a 4-point scale ranging from 0 (not at all) to 3 (nearly every day). Total scores range from 0 (no symptoms) to 27 (severe symptoms). Scores > 9 are indicative of a clinically significant level of depressive symptoms (i.e. diagnosis of depression). The measure demonstrates sound psychometric properties [31], has been used extensively to measure treatment outcomes during iCBT interventions targeting depression and anxiety [25], and has been validated in pregnancy and postpartum samples [35, 36].

*Generalized Anxiety Disorder 7 item scale* (GAD-7) [30]. The GAD-7 is a seven-item self-report measure of general anxiety symptoms experienced over the past two weeks. The GAD-7 is based on DSM-IV criteria for generalised anxiety disorder (GAD). Items (e.g. ‘Feeling nervous, anxious, or on edge’) are rated on a 4-point scale ranging from 0 (not at all) to 3 (nearly every day). Total scores are in the range of 0–21, with scores > 9 indicating the possibility of an anxiety disorder. The GAD-7 has been validated for diagnosing GAD in the general population and demonstrates good internal consistency and test–retest reliability [30, 37]. The GAD-7 has been validated for identifying GAD in a sample of pregnant women, with a cut-off score of 13 demonstrating a sensitivity of 61% and specificity of 73% [38].

#### Secondary outcomes

*Kessler-10 psychological distress scale* (K10) [33]*.* The K10 is a ten-item self-report measure designed to assess general (i.e. non-specific) psychological distress over the past 30 days. For the current RCTs, the time frame was modified to assess distress in the past two weeks. Items (e.g. ‘About how often did you feel hopeless?’) are rated on a 5-point scale ranging from 1 (none of the time) to 5 (all of the time). Total scores range from 10 (no distress) to 50 (severe distress). The K10 demonstrates strong psychometric properties in non-perinatal samples [39] and is advantageous for monitoring iCBT patients [40]. The K10 has also demonstrated acceptable sensitivity and specificity in identifying depression and some anxiety disorders in pregnant women [41].

*Edinburgh Postnatal Depression Scale* (EPDS) [42]. The EPDS is a ten-item self-report screening tool originally developed to detect symptoms of postpartum depression, although it is also used to assess for depression during pregnancy [43]. The EPDS enquires as to the intensity of symptoms over the past seven days (e.g. ‘I have felt sad or miserable’; ‘I have blamed myself unnecessarily when things went wrong’). Items are rated on a scale from 0 (e.g. no, not at all) to 3 (e.g. yes, most of the time), with a maximum total score of 30. A total score of > 12 is indicative of possible depression. The EPDS is well validated as a screening tool in antenatal and postpartum samples, with sensitivity in the range of 70–85% and specificity in the range of 77–85% in identifying a major depressive episode [20, 44, 45].

*World Health Organization Quality of Life* (WHOQOL-BREF) [46]. The WHOQOL-BREF is a 26-item self-report measure assessing quality of life (QOL) over the past four weeks. QOL is measured across four domains: physical health (e.g. sleep, pain); psychological health (e.g. self-esteem, concentration); social relationships (e.g. support; personal relationships); and environment (e.g. physical safety; financial resources). Items (e.g. ‘How much do you enjoy life?’) are rated on a 5-point Likert scale ranging from 1 (e.g. not at all) to 5 (e.g. an extreme amount). The WHOQOL-BREF has been validated in postpartum women, with satisfactory internal consistency and high discriminant validity [47]. The computerised version of the WHOQOL-BREF has demonstrated excellent psychometric properties [48].

*Maternal Antenatal Attachment Scale* (MAAS) [49]. The MAAS is a 19-item self-report measure designed to assess maternal feelings of emotional bonding to the fetus over the past two weeks. The scale has two domains indicating quality of maternal bonding (i.e. affective experiences such as closeness, tenderness); and intensity of preoccupation with the fetus (i.e. amount of time spent thinking about the unborn baby). All items are rated on a 5-point Likert scale ranging from 1 (low attachment) to 5 (high attachment). Total scores are in the range of 19–95, with higher scores indicative of better maternal attachment. The MAAS demonstrates acceptable internal consistency and construct validity in pregnant women [49, 50].

*Maternal Postnatal Attachment Scale* (MPAS) [51]. The MPAS is a 19-item self-report measure designed to assess maternal feelings of emotional bonding to the infant over the past two weeks. The scale has three domains indicating quality of attachment, absence of hostility towards the infant and pleasure in interacting with the infant. Items (e.g. ‘Over the last two weeks I would describe my feelings for the baby as…’) are rated on 2-, 3-, 4- and 5-point scales, with responses recoded to represent a score of 1 (low attachment, e.g. dislike) to 5 (high attachment, e.g. intense affection). Higher total scores are indicative of better maternal attachment. The MPAS has demonstrated acceptable levels of internal consistency, test–retest reliability and construct validity in postnatal women [51–53].

*Karitane Parenting Confidence Scale* (KPCS) [54]. The KPCS is a 15-item self-report questionnaire designed to measure perceived parental self-efficacy in parents with infants aged 0 and 12 months. ‘Task-specific’ items (e.g. ‘I can settle my baby’; ‘I am confident about playing with my baby’) are rated on a 4-point scale ranging from 0 (no, hardly ever) to 3 (yes, most of the time), with higher scores indicating higher parenting confidence. Total scores < 40 are indicative of lower than normal parenting confidence. The KPCS has been validated for use with mothers and demonstrates acceptable test–retest reliability (*r* = 0.88), internal consistency (Cronbach’s *a* = 0.81) and convergent and discriminant validity [54].

*Beck Depression Inventory, second edition* (BDI-II) [32]. The BDI-II is a 21-item self-report measure designed to assess the presence and severity of depressive symptom over the past two weeks and is based on DSM-IV criteria for MDD. Items are rated on a 3-point scale, with higher scores indicating more severe depression. The BDI-II possesses high internal consistency for psychiatric and non-psychiatric populations [32]. Only Question 9 (‘Suicidal thoughts or wishes: (0) I don’t have any thoughts of killing myself; (1) I have thoughts of killing myself, but I would not carry them out; (2) I would like to kill myself; (3) I would kill myself if I had the chance’) will be used to help determine suicidal ideation and suicide risk.

##### Treatment-relevant outcomes

Two items from the Credibility/Expectancy Questionnaire (CEQ) [55] will be used to assess expectancy of treatment benefit (e.g. ‘At this point, how logical does the program offered to you seem?’; ‘At this point, how successfully do you think this treatment will be in reducing your anxiety and/or depression symptoms?’). Items are rated on a 0 (not at all) to 9 (very much) scale and will be completed before Lesson 1 and Lesson 2. Treatment satisfaction will be assessed with 19 questions derived from the Treatment Satisfaction Questionnaire (TSQ) [56]. Items will enquire into the perceived helpfulness of the program (e.g. ‘How satisfied are you with the skills that this program has taught you to manage your symptoms of anxiety and/or depression?’; ‘What did you find most helpful/unhelpful?’; ‘How logical was the program?’), relatedness with characters (‘How much did you relate to the character Lee?’) and additional program feedback (e.g. ‘What could we do to improve the *MUMentum* program?’). Treatment adherence will be reflected in the mean number of CBT sessions viewed.

#### Description of iCBT programs

*MUMentum* was adapted from a six-lesson, guided iCBT program for anxiety and depression in the general population [25, 57]. Given women face many barriers to treatment engagement (e.g. time-poor; lack of treatment relevance to perinatal-related issues), the six-lesson transdiagnostic CBT program was divided into two brief courses focused on the antenatal or postpartum period with content presented over three lessons. Both *MUMentum* programs cover the same core principles and skills of CBT including psychoeducation, cognitive restructuring and problem-solving, behavioural activation and relapse prevention, with each lesson building upon the previous lesson (see Table 1). However, both courses differ in the context in which CBT skills are learned. The pregnancy course (*MUMentum Pregnancy*) is specifically tailored to women in the antenatal period and focuses on learning CBT skills in the context of character experiences, challenges and symptoms common during pregnancy (e.g. difficulties conceiving; increased anxiety surrounding the impending birth and health of the baby). In comparison, the postpartum course (*MUMentum Postnatal*) has been developed to target the unique issues, challenges and symptoms common during the postpartum period (e.g. asking for help; busting myths about motherhood; adjusting to changes in relationships; prioritising self-care; intrusive thoughts).

**Table 1:** Core CBT skills of *MUMentum* programs

Lesson	Skills	Extra resources
1	• Psychoeducation: o About anxiety and depression o Identifying symptoms o Cognitive behavioural model o Prioritising self-care o Physical symptoms o Partners and supporters • Controlled breathing • Progressive muscle relaxation	• Medication for anxiety and depression during pregnancy and breastfeeding • Sleep hygiene • Fight-or-flight response • Pleasant activities • FAQs • Further skill examples
2	• Psychoeducation: o About thoughts o Identifying unhelpful thoughts o Shifting unhelpful thoughts o Accepting uncertainty • Thought challenging • Coping cards • Structured problem-solving	• Understanding intrusive thoughts and images • FAQs • Further skill examples
3	• Psychoeducation: o Unhelpful behaviours (low activity; avoidance) o Facing your fears • Activity planning and monitoring • Graded exposure • Assertive communication • Relapse prevention	• Self-care plan • FAQs • Further skill examples

Both programs are aimed as stand-alone, psychoeducational courses to introduce women to CBT skills to help manage symptoms of anxiety and depression. Content for each program is presented in the form of an illustrated story in which two fictional characters, who experience anxiety and depression, gain mastery over their symptoms with the help of a clinician (i.e. psychologist). Character stories for each program were developed with the key aim of being generalisable to a broad range of women and common experiences during pregnancy or postpartum (e.g. miscarriage; difficulties becoming pregnant; previous postpartum depression; traumatic birth, partner difficulties). Each lesson consists of: (1) a set of lesson slides depicting the characters story and use of CBT techniques (e.g. activity planning); (2) a lesson summary and action plan to review and implement the key topics and skills demonstrated in the slides; (3) and a range of supplementary resources (e.g. good sleep guide). A library of general resources is also available for participants to refer to throughout the program that cover additional information (e.g. emergency contacts; understanding medications for anxiety and depression during pregnancy and breastfeeding; information for partners and supporters) and further examples of key skills.

### Data management

All data will be collected and stored via the Virtual Clinic server. Any identifiable information collected remains confidential. Only members of the site (CRUfAD) research team will have access to participant information and identifiable data in order to monitor participant progress.

During data analysis, re-identifiable data (i.e. coded data) will be used. At study completion, non-identifiable data will be written to a password-protected database. All data will be extracted from the Virtual Clinic servers in the form of an Excel-compatible file to be transferred to the IBM Statistical Package for the Social Sciences (IBM SPSS, IBM Corp., Armonk, NY, USA) by a member of the research team.

Participants will be informed during the consent process at application that the research team plans to disseminate the trial results in peer-reviewed scientific publications and presentations. Participants are informed that in any such dissemination, their anonymity will be maintained. Participants will be sent (via email) a written summary of the results in lay terms following completion of the trial study phase.

### Statistical Methods

As there is limited research outlining the efficacy of iCBT for the treatment of perinatal anxiety and depression, power calculation of sample size (using ClinTools software) was informed by a published RCT of a transdiagnostic iCBT program [25]. To detect a between-group effect corresponding to Hedges’ *g* of 0.80, the minimum sample size for each group (alpha set at 0.05, power at 0.80) was identified as 25 per group. At least 10% more will be recruited to account for expected attrition.

All analyses will be conducted at conclusion of the trial when the desired sample size has been obtained. All analyses will be conducted using Statistical Package for the Social Sciences (IBM SPSS, IBM Corp., Armonk, NY, USA). Significance testing of group differences regarding demographic data and pre-treatment measurements will be conducted using analysis of variance (ANOVA) and Chi square (χ2) where the variables consist of nominal (or categorical) data.

Intent-to-treat (ITT) mixed models using restricted maximum likelihood (REML) estimation will be used to account for missing data due to participant dropout. Mixed models do not assume that the last measurement is stable (the last observation carried forward assumption) [58]. REML models are appropriate for RCTs with multiple time-points and pre- to post-only designs [59]. The assumption that data are missing at random will be evaluated using binary logistic regression to predict dropout and by comparing these two groups (those with missing data to those with complete data) on baseline measures. Significant effects will be followed up with pairwise contrasts comparing mean post-treatment (and follow-up) scores between groups. Effect sizes will be calculated between groups (Hedges’ *g* to account for small sample size) and within groups (Cohen’s *d*, adjusting for the repeated measures correlation) using the pooled standard deviation and adjusted for sample size. Post-hoc exploratory mediation analyses, using the PROCESS syntax for SPSS PROCESS [60], will be used to examine the associations between symptom change and changes in the maternal factors of mother–infant bonding, parenting confidence and maternal quality of life.

## Discussion

The current RCTs will evaluate the efficacy of an iCBT intervention for maternal symptoms of anxiety, depression and overall psychological distress during the antenatal (study 1) and postpartum period (study 2). If proven efficacious and acceptable, the *MUMentum* programs will provide women in Australia with an evidence-based, easily accessible, psychoeducational CBT intervention in which to manage symptoms of perinatal anxiety, depression and distress. Results from the proposed RCTs will also contribute to the growing area of perinatal e-health interventions and aim to address several limitations in the literature.

Specifically, the *MUMentum* programs will be the first Internet-delivered interventions targeted at symptoms of perinatal anxiety. Perinatal anxiety is a common and disabling condition in women during the perinatal period, is highly co-morbid with depression, yet is often underdiagnosed and undertreated [38]. To date, no Internet-delivered interventions have targeted symptoms of anxiety (with or without depressive symptoms) during the perinatal period.

Treatment for depression during the antenatal period has also received limited attention in comparison to postpartum depression. The *MUMentum Pregnancy* program will be the second iCBT intervention developed specifically for women in the antenatal period [19]. Given that antenatal depression and anxiety are robust predictors of postpartum depression, our evaluation of the impact of these programs on mother and infant outcomes (e.g. mother–infant bonding; parenting confidence; quality of life) will contribute to our understanding of how iCBT can be tailored to maximise the benefit to both mothers and infants.

In comparison to the therapist-guided interventions that have recently been evaluated [17–19], the *MUMentum* programs will be the first brief (i.e. three sessions) unguided iCBT interventions for perinatal women that do not require clinician support or coaching. If efficacious, the *MUMentum* programs have the potential to be easily disseminated via https://thiswayup.org.au/ to large numbers of women across Australia as a ‘first step’ intervention in a stepped care model of treatment (i.e. for women screening positive for anxiety, depressive or distress symptoms during pregnancy or postpartum). Future research should aim to investigate the cost-effectiveness of such interventions in primary care and the impact of varying levels of clinician guidance and coaching on patient outcomes.

While the current RCTs aim to address gaps in the perinatal e-health literature, the following potential limitations are noted. First, the control group in the current RCTs are TAU control, rather than an active control condition. It is thus difficult to control for usual antenatal and postpartum care that participants receive throughout the trial. It is also not possible to compare the efficacy of a perinatal-specific iCBT intervention to a general iCBT intervention that is not specifically developed for women in the perinatal period (e.g. general depression program), other treatment approach or modality (e.g. face-to-face) as an active control condition was not included. Future research should investigate the efficacy of online-delivered interventions in comparison to active control conditions and face-to-face psychological therapy. It is also important to determine what treatment factors are most effective for which patients and whether the presence of anxiety or depressive disorders, or the severity of anxiety or depressive symptoms, influences treatment response in addition to potential side effects of the intervention [61].

Second, the follow-up period for this study was limited to four weeks post treatment. Previous research in this area that has employed longer follow-up periods (e.g. three months) have reported high attrition (i.e. dropout) (e.g. O’Mahen et al. [62]). We therefore aimed to keep the follow-up period as short as possible, particularly for study 1 in which we aimed to evaluate follow-up treatment outcomes while women were still pregnant. Future research evaluating antenatal interventions would benefit from inclusion of a postpartum follow-up, given antenatal anxiety and depression are strong predictors of postpartum depression [27, 28]. It is necessary for future research to examine long-term outcomes to determine whether positive outcomes are sustained over a longer duration beyond completion of treatment, particularly from pregnancy through to postpartum. This information would inform how antenatal treatment interventions can be tailored most effectively to prevent postpartum anxiety and depression.

Third, both RCTs will recruit women who meet the required threshold to be considered experiencing ‘clinically elevated’ anxiety and/or depression on self-report measures, rather than a formally assessed sample who meet criteria for a diagnosis of an anxiety or depressive disorder. It will therefore be difficult to generalise findings from this study due to the small sample size and reliance on self-report data only. Future research would benefit from investigating treatment interventions in larger randomised controlled studies utilising both self-report symptom measures and clinician-administered diagnostic measures. However, it is important to note that while clinical diagnostic interviews are the “gold standard” of measuring mental health problems, research suggests that diagnostic criteria may not account for all perinatal-specific problems or difficulties [63]. As a substantial proportion of perinatal women will not fulfil all diagnostic criteria yet will experience similar levels of distress and disability [64], we aimed to develop the *MUMentum* programs for women experiencing clinically significant symptoms of anxiety and/or depression and evaluate the programs’ efficacy in this target sample. While this study aims to establish the efficacy of the programs in reducing symptom severity in a clinical sample, future research would benefit from investigating the effectiveness of the *MUMentum* programs in the ‘end users’ of the program (i.e. those screening positive for anxiety, depression or distress) in primary care effectiveness trials.

Given the prevalence of perinatal anxiety and depression and the negative outcomes for both the mother and child, the development of evidence-based accessible treatments that target both anxiety and depressive symptoms, and overall psychological distress is imperative. Simple, brief interventions that do not rely on mental health specialists and can thus be more widely disseminated is an important area of focus for clinicians and researchers (Additional file 1).

### Trial status

The first patient was enrolled 5 October 2016. To date, 98 participants have met eligibility requirements and been randomised to either study 1 or study 2. Recruitment is ongoing. Data collection aims to be complete in February 2018.

## Additional file

Additional file 1: SPIRIT 2013 Checklist: Recommended items addressed in clinical trial protocol. (DOC 120 kb)

## Abbreviations

BDI-II: Beck Depression Inventory, 2nd edition
CRUfAD: Clinical Research Unit for Anxiety and Depression
DSM-IV: Diagnostic Statistical Manual, 4th edition
EPDS: Edinburgh Postnatal Depression Scale
GAD-7: Generalised Anxiety Disorder, 7-item scale
iCBT: Internet-delivered cognitive behavioural therapy
K10: Kessler-10 Psychological Distress Scale
KPCS: Karitane Parenting Confidence Scale
MAAS: Maternal Antenatal Attachment Scale
MDD: Major depressive disorder
MPAS: Maternal Postnatal Attachment Scale
PHQ-9: Patient health questionnaire, 9-item
RCT: Randomised controlled trial
TAU: Treatment as usual control
WHOQOL-BREF: World Health Organization Quality of Life Scale

## Footnotes

1 Study 2 will be conducted in an updated online trials system which allows for automatic randomisation after application. This system is not available for study 1.

## References

[1] Woolhouse H, Brown S, Krastev A, Perlen S, Gunn J (2009). Seeking help for anxiety and depression after childbirth: results of the Maternal Health Study. Arch Womens Ment Health..

[2] Biaggi A, Conroy S, Pawlby S, Pariante CM (2016). Identifying the women at risk of antenatal anxiety and depression: A systematic review. J Affect Disord..

[3] Dennis CL, Falah-Hassani K, Shiri R (2017). Prevalence of antenatal and postnatal anxiety: Systematic review and meta-analysis. Br J Psychiatry..

[4] O’Hara MW, Wisner KL (2014). Perinatal mental illness: Definition, description and aetiology. Best Pract Res Clin Obstet Gynaecol..

[5] Austin M-P, Frilingos M, Lumley J, Hadzi-Pavlovic D, Roncolato W, Acland S (2008). Brief antenatal cognitive behaviour therapy group intervention for the prevention of postnatal depression and anxiety: a randomised controlled trial. J Affect Disord..

[6] Goodman JH, Tyer-Viola L (2010). Detection, treatment, and referral of perinatal depression and anxiety by obstetrical providers. J Women’s Health..

[7] Dennis CL, Chung‐Lee L (2006). Postpartum depression help‐seeking barriers and maternal treatment preferences: A qualitative systematic review. Birth..

[8] Goodman JH (2009). Women’s attitudes, preferences, and perceived barriers to treatment for perinatal depression. Birth..

[9] Kopelman RC, Moel J, Mertens C, Stuart S, Arndt S, O’Hara MW (2008). Barriers to care for antenatal depression. Psychiatr Serv..

[10] Kingston D, Austin M-P, Heaman M, McDonald S, Lasiuk G, Sword W (2015). Barriers and facilitators of mental health screening in pregnancy. J Affect Disord..

[11] Stein A, Pearson RM, Goodman SH, Rapa E, Rahman A, McCallum M (2014). Effects of perinatal mental disorders on the fetus and child. Lancet..

[12] O’Connor TG, Heron J, Golding J, Beveridge M, Glover V (2002). Maternal antenatal anxiety and children’s behavioural/emotional problems at 4 years. Br J Psychiatry..

[13] Glasheen C, Richardson GA, Fabio A (2010). A systematic review of the effects of postnatal maternal anxiety on children. Arch Womens Ment Health..

[14] Grote NK, Bridge JA, Gavin AR, Melville JL, Iyengar S, Katon WJ (2010). A meta-analysis of depression during pregnancy and the risk of preterm birth, low birth weight, and intrauterine growth restriction. Arch Gen Psychiatry..

[15] Andrews G, Cuijpers P, Craske MG, McEvoy P, Titov N (2010). Computer therapy for the anxiety and depressive disorders is effective, acceptable and practical health care: a meta-analysis. PLoS One..

[16] Hedman E, Ljótsson B, Kaldo V, Hesser H, El Alaoui S, Kraepelien M (2014). Effectiveness of Internet-based cognitive behaviour therapy for depression in routine psychiatric care. J Affect Disord..

[17] Milgrom J, Danaher BG, Gemmill AW, Holt C, Holt CJ, Seeley JR (2016). Internet cognitive behavioral therapy for women with postnatal depression: a randomized controlled trial of MumMoodBooster. J Med Internet Res.

[18] Pugh NE, Hadjistavropoulos HD, Dirkse D (2016). A randomised controlled trial of therapist-assisted, internet-delivered cognitive behavior therapy for women with maternal depression. PLoS One..

[19] Forsell E, Bendix M, Holländare F, von Schultz BS, Nasiell J, Blomdahl-Wetterholm M (2017). Internet delivered cognitive behavior therapy for antenatal depression: a randomised controlled trial. J Affect Disord..

[20] Grigoriadis S, de Camps MD, Barrons E, Bradley L, Eady A, Fishell A (2011). Mood and anxiety disorders in a sample of Canadian perinatal women referred for psychiatric care. Arch Womens Ment Health..

[21] Harvey AG (2004). Cognitive behavioural processes across psychological disorders: A transdiagnostic approach to research and treatment.

[22] Matthey S, Barnett B, Howie P, Kavanagh DJ (2003). Diagnosing postpartum depression in mothers and fathers: whatever happened to anxiety?. J Affect Disord..

[23] Heron J, O’Connor TG, Evans J, Golding J, Glover V (2004). The course of anxiety and depression through pregnancy and the postpartum in a community sample. J Affect Disord..

[24] McEvoy PM, Nathan P, Norton PJ (2009). Efficacy of transdiagnostic treatments: A review of published outcome studies and future research directions. J Cogn Psychother..

[25] Newby JM, Mackenzie A, Williams AD, McIntyre K, Watts S, Wong N (2013). Internet cognitive behavioural therapy for mixed anxiety and depression: a randomized controlled trial and evidence of effectiveness in primary care. Psychol Med..

[26] Titov N, Dear BF, Schwencke G, Andrews G, Johnston L, Craske MG (2011). Transdiagnostic internet treatment for anxiety and depression: a randomised controlled trial. Behav Res Ther..

[27] Robertson E, Grace S, Wallington T, Stewart DE (2004). Antenatal risk factors for postpartum depression: a synthesis of recent literature. Gen Hosp Psychiatry..

[28] Milgrom J, Gemmill AW, Bilszta JL, Hayes B, Barnett B, Brooks J (2008). Antenatal risk factors for postnatal deperssion: A large prospective study. J Affect Disord..

[29] Schulz KF, Altman DG, Moher D (2010). CONSORT 2010 statement: updated guidelines for reporting parallel group randomised trials. BMC Med..

[30] Spitzer RL, Kroenke K, Williams JB, Löwe B (2006). A brief measure for assessing generalized anxiety disorder: the GAD-7. Arch Intern Med..

[31] Kroenke K, Spitzer R, Williams J (2001). The PHQ-9: validity of a brief depression severity measure [Electronic version]. J Gen Intern Med..

[32] Beck AT, Steer RA, Brown GK (1996). Beck depression inventory - manual.

[33] Kessler RC, Andrews G, Colpe LJ, Hiripi E, Mroczek DK, Normand S-L (2002). Short screening scales to monitor population prevalences and trends in non-specific psychological distress. Psychol Med..

[34] American Psychiatric Association (2000). Diagnostic and statistical manual of mental disorders (text revision).

[35] Sidebottom AC, Harrison PA, Godecker A, Kim H (2012). Validation of the Patient Health Questionnaire (PHQ)-9 for prenatal depression screening. Arch Womens Ment Health..

[36] Gjerdingen D, Crow S, McGovern P, Miner M, Center B (2009). Postpartum depression screening at well-child visits: validity of a 2-question screen and the PHQ-9. Ann Fam Med..

[37] Löwe B, Decker O, Müller S, Brähler E, Schellberg D, Herzog W (2008). Validation and standardization of the Generalized Anxiety Disorder Screener (GAD-7) in the general population. Med Care..

[38] Simpson W, Glazer M, Michalski N, Steiner M, Frey BN (2014). Comparative efficacy of the generalized anxiety disorder 7-item scale and the Edinburgh Postnatal Depression Scale as screening tools for generalized anxiety disorder in pregnancy and the postpartum period. Can J Psychiatry..

[39] Furukawa TA, Kessler RC, Slade T, Andrews G (2003). The performance of the K6 and K10 screening scales for psychological distress in the Australian National Survey of Mental Health and Well-Being. Psychol Med..

[40] Sunderland M, Wong N, Hilvert-Bruce Z, Andrews G (2012). Investigating trajectories of change in psychological distress amongst patients with depression and generalised anxiety disorder treated with internet cognitive behavioural therapy. Behav Res Ther..

[41] Spies G, Stein D, Roos A, Faure S, Mostert J, Seedat S (2009). Validity of the Kessler 10 (K-10) in detecting DSM-IV defined mood and anxiety disorders among pregnant women. Arch Womens Ment Health..

[42] Cox JL, Holden JM, Sagovsky R (1987). Detection of postnatal depression: Development of the 10-item Edinburgh Postnatal Depression Scale. Br J Psychiatry..

[43] Cox JL, Chapman G, Murray D, Jones P (1996). Validation of the Edinburgh Postnatal Depression Scale (EPDS) in non-postnatal women. J Affect Disord..

[44] Navarro P, Ascaso C, Garcia-Esteve L, Aguado J, Torres A, Martín-Santos R (2007). Postnatal psychiatric morbidity: a validation study of the GHQ-12 and the EPDS as screening tools. Gen Hosp Psychiatry..

[45] Bergink V, Kooistra L, den Lambregtse-van Berg MP, Wijnen H, Bunevicius R, van Baar A (2011). Validation of the Edinburgh Depression Scale during pregnancy. J Psychosom Red..

[46] Skevington SM, Lotfy M, O’Connell KA (2004). The World Health Organization’s WHOQOL-BREF quality of life assessment: psychometric properties and results of the international field trial. A report from the WHOQOL group. Qual Life Res.

[47] Webster J, Nicholas C, Velacott C, Cridland N, Fawcett L (2010). Validation of the WHOQOL‐BREF among women following childbirth. Aust NZ J Obstet Gynaecol..

[48] Chen W-C, Wang J-D, Hwang J-S, Chen C-C, Wu C-H, Yao G (2009). Can the web-form WHOQOL-BREF be an alternative to the paper-form?. Soc Indicators Res..

[49] Condon JT (1993). The assessment of antenatal emotional attachment: development of a questionnaire instrument. Psychol Psychother-T..

[50] Condon JT, Corkindale C (1997). The correlates of antenatal attachment in pregnant women. Psychol Psychother-T..

[51] Condon JT, Corkindale CJ (1998). The assessment of parent-to-infant attachment: development of a self-report questionnaire instrument. J Reprod Infant Psychol..

[52] Van Bussel JC, Spitz B, Demyttenaere K (2010). Three self-report questionnaires of the early mother-to-infant bond: reliability and validity of the Dutch version of the MPAS, PBQ and MIBS. Arch Womens Ment Health..

[53] Scopesi A, Viterbori P, Sponza S, Zucchinetti P (2004). Assessing mother‐to‐infant attachment: the Italian adaptation of a self‐report questionnaire. J Reprod Infant Psychol..

[54] Črnčec R, Barnett B, Matthey S (2008). Development of an instrument to assess perceived self‐efficacy in the parents of infants. Res Nurs Health..

[55] Devilly GJ, Borkovec TD (2000). Psychometric properties of the credibility/expectancy questionnaire. J Behav Ther Exp Psychiatry..

[56] Cox BJ, Fergus KD, Swinson RP (1994). Patient satisfaction with behavioral treatments for panic disorder with agoraphobia. J Anxiety Disord..

[57] Newby JM, Mewton L, Williams AD, Andrews G (2014). Effectiveness of transdiagnostic internet cognitive behavioural treatment for mixed anxiety and depression in primary care. J Affect Disord..

[58] Gueorguieva R, Krystal JH (2004). Move over ANOVA: progress in analyzing repeated-measures data and its reflection in papers published in the archives of general psychiatry. Arch Gen Psychiatry..

[59] Salim A, Mackinnon A, Christensen H, Griffiths K (2008). Comparison of data analysis strategies for intent-to-treat analysis in pre-test–post-test designs with substantial dropout rates. Psychiatry Res..

[60] Hayes AF (2013). Introduction to mediation, moderation, and conditional process analysis: A regression-based approach.

[61] Goodman JH, Watson GR, Stubbs B (2016). Anxiety disorders in postpartum women: A systematic review and meta-analysis. J Affect Disord..

[62] O’Mahen HA, Woodford J, McGinley J, Warren FC, Richards DA, Lynch TR (2013). Internet-based behavioral activation—Treatment for postnatal depression (Netmums): A randomized controlled trial. J Affect Disord..

[63] Ayers S, Coates R, Matthey S (2015). Identifying perinatal anxiety.

[64] Wenzel A, Haugen EN, Jackson LC, Brendle JR (2005). Anxiety symptoms and disorders at eight weeks postpartum. J Anxiety Disord..

